# Who would like a monster like me to be alive? Obsessive compulsive disorder or pedophilia in a patient with high functioning autism spectrum disorder

**DOI:** 10.1192/j.eurpsy.2021.624

**Published:** 2021-08-13

**Authors:** C. Pastor Jordá, A. Gu, A. Kelly, D. Mckessy, S. Shear

**Affiliations:** 1 Child And Adolescent Psychiatry, Alicia Koplowitz Foundation, Madrid, Spain; 2 Child And Adolescent Psychiatry, UPMC Western Psychiatric Hospital, Pittsburgh, United States of America

**Keywords:** ASD, Pedophilia, ocd

## Abstract

**Introduction:**

Case of a 17yo patient with high functioning ASD and OCD with obsessions about being a pedophile, with suicidal ideation and self-harming behaviors. He was followed in outpatient care for one year since his first contact with Mental Health, following an inpatient admission for suicidal ideation.

**Objectives:**

Differential diagnosis between OCD, ASD and possible pedophilia. Learn about different levels of care involved, and other possibilities. Therapy resources used.

**Methods:**

Description of the case report: description of initial and final Mental Status Exam Differential Diagnosis: ASD vs OCD vs Pedophilia vs Depressive Disorder Children’s Yale-Brown Obsessive Compulsive Scale Therapy: family based therapy, and Exposure response prevention therapy.

**Results:**

Intrusive images, and reassurance seeking, helped with OCD diagnosis. ASD made symptoms harder to manage with SSRIs alone, which drove to add Aripiprazol at low doses in outpatient care. CY-BOCS showed obsessions other than doubts about being a pedophile. He participated in Exposure response prevention therapy with response, especially when antipsychotic medication was added. Family based therapy worked with his parents in not providing excessive reassurance, and with the patient in gaining insight about his OCD. Decreased anxiety, decreased self-deprecation and no new suicidal thoughts Functionality of the patient in the community improved, with possibility of going college next year.

**Conclusions:**

Recommendation of good assessment of sexuality in ASD population Importance of individual and family therapy for OCD and specially when there is poor judgement and insight in the patient. Importance of combined treatment: pharmacology + therapy

**Conflict of interest:**

Alicia Koplowitz Foundation

